# Clinical significance of the expression of autophagy-associated marker, beclin 1, in breast cancer patients who received neoadjuvant endocrine therapy

**DOI:** 10.1186/s12885-016-2270-9

**Published:** 2016-03-16

**Authors:** Takayuki Ueno, Shigehira Saji, Masahiro Sugimoto, Norikazu Masuda, Katsumasa Kuroi, Nobuaki Sato, Hiroyuki Takei, Yutaka Yamamoto, Shinji Ohno, Hiroko Yamashita, Kazufumi Hisamatsu, Kenjiro Aogi, Hiroji Iwata, Shigeru Imoto, Hironobu Sasano, Masakazu Toi

**Affiliations:** Department of Breast Surgery, Kyoto University Hospital, Kyoto, Japan; Department of Breast Surgery, Kyorin University Hospital, 6-20-2 Shinkawa Mitaka, 181-8611 Tokyo, Japan; Department of Target Therapy Oncology, Kyoto University Graduate School of Medicine, Kyoto, Japan; Institute for Advanced Biosciences Keio University, Yamagata, Japan; National Hospital Organization Osaka National Hospital, Osaka, Japan; Tokyo Metropolitan Cancer and Infectious Diseases Center Komagome Hospital, Tokyo, Japan; Niigata Cancer Center Hospital, Niigata, Japan; Division of Breast Surgery, Saitama Cancer Center, Saitama, Japan; Department of Breast and Endocrine Surgery, Kumamoto University, Kumamoto, Japan; National Hospital Organization Kyushu Cancer Center, Fukuoka, Japan; Breast and Endocrine Surgery, Hokkaido University Hospital, Sapporo, Japan; Hiroshima City Asa Hospital, Hiroshima, Japan; National Hospital Organization Shikoku Cancer Center, Ehime, Japan; Aichi Cancer Center Hospital, Nagoya, Japan; Tohoku University School of Medicine, Sendai, Japan

**Keywords:** Beclin 1, Autophagy, Ki-67, Breast cancer, Aromatase inhibitor, Neoadjuvant endocrine therapy

## Abstract

**Background:**

Neoadjuvant endocrine therapy (NAE) has been employed to improve surgical outcomes for hormone receptor-positive breast cancers in postmenopausal women. Endocrine responsiveness is estimated by expressions of hormone receptors, but its heterogeneity has been recognized. Autophagy is an evolutionally conserved process associated with cell survival and cell death and has been implicated in cancer treatment.

**Methods:**

In order to examine the possible association between autophagy and response to endocrine therapy, we evaluated the status of autophagy-associated markers, beclin 1 and LC3, and apoptosis-associated markers, TUNEL and M30, in pre- and post-treatment specimens from 71 patients in a multicenter prospective study of neoadjuvant exemestane (JFMC34-0601).

**Results:**

Immunoreactivity of the autophagy-associated markers, beclin 1 and LC3, in carcinoma cells increased in 14 % and 52 % of the patients, respectively, following the exemestane treatment. These increases were statistically significant (beclin 1, *p* = 0.016, *N* = 49; LC3, *p* < 0.0001, *N* = 33). The status of M30 immunoreactivity decreased (*p* = 0.008, *N* = 47) and TUNEL remained unchanged (*N* = 53). In addition, tumors with pre-treatment stromal beclin 1 immunoreactivity revealed poor clinical and pathological responses compared with those without stromal beclin 1 immunoreactivity (25 % vs 67 % for clinical response, *p* = 0.011, *N* = 51; 0 % vs 41 % for pathological response, *p* = 0.0081, *N* = 49). Tumors with positive pre-treatment stromal beclin 1 had a higher baseline Ki-67 labeling index (both hot spot and overall average) than those without (*p* = 0.042 and 0.0075, respectively, *N* = 53). Results of logistic regression analyses revealed that stromal beclin 1 was a predictor for clinical and pathological responses while ER, PR, Ki-67, and stromal LC3 expressions were not.

**Conclusions:**

Results of our present study demonstrated that beclin 1 and LC3 immunoreactivity increased in carcinoma cells following exemestane treatment and that the status of pre-treatment stromal beclin 1 is associated with higher carcinoma cell proliferation and poor clinical and pathological responses to NAE.

**Trial registration:**

UMIN C000000345 (2006/03/06)

**Electronic supplementary material:**

The online version of this article (doi:10.1186/s12885-016-2270-9) contains supplementary material, which is available to authorized users.

## Background

Neoadjuvant endocrine therapy (NAE) is one of the treatment options for postmenopausal patients with hormone receptor (HR)-positive breast cancers. NAE increases the breast-conserving rate, which is indeed one of its major purposes [1]. ACOSOG Z1031, a study comparing three neoadjuvant aromatase inhibitors, has reported clinical response rates ranging from 62 to 75 % and progressive disease (PD) rates from 4.7 to 7.3 % [2]. It has therefore become clinically important to predict endocrine responsiveness in order to improve clinical outcomes for HR-positive breast cancers.

Elucidation of cellular responses to endocrine treatment is pivotal for understanding endocrine responsiveness. Endocrine treatment does reduce breast cancer volume in almost 70 % patients but apoptosis was also reported not to be necessarily increased in carcinoma cells following endocrine treatment [3]. Therefore, it remains to be determined how carcinoma cells react to endocrine therapy and how clinically detected volume reduction is accomplished.

Autophagy is one of the mechanisms of cell death and represents an evolutionary conserved process critical for adaptation to cellular stress and maintenance of cellular homeostasis. During autophagy, long-lived proteins and organelles are degraded by lysosomal degradation mechanisms. Autophagy is implicated in a dual role in cancer biology: tumor suppression and promotion [4]. Monoallelic deletion of the autophagy-related gene, beclin 1, was known to increase the incidence of spontaneous tumors in lung, liver and lymphoid tissue, suggestive of an anti-tumor effect of autophagy [5]. Consistently, breast cancer tissues were reported to express less beclin 1 than normal breast tissues [6]. In contrast, autophagy has been reported to be required for Ras-mediated transformation of non-malignant breast epithelial cells [7], and abrogation of autophagy to result in decreased tumor growth, indicating a pro-tumor effect of autophagy [8]. It is therefore pivotal to determine how autophagy is involved in the treatment responses of breast carcinoma cells.

In this study, we therefore investigated the status of autophagy-associated markers, beclin 1 and LC3, in conjunction with apoptosis-associated markers, TUNEL and M30, in breast cancer tissues. We used archived specimens, both pre- and post-treatment cases, from a neoadjuvant exemestane study (JFMC34-0601), in order to examine the association of these markers with clinical and pathological responses to endocrine treatment. Results revealed that endocrine treatment increased the expression status of autophagy-associated markers in carcinoma cells. In addition, the status of pre-treatment stromal beclin 1 was significantly associated with poor clinical and pathological responses of the patients to endocrine treatment.

## Methods

**Fig. 1:**
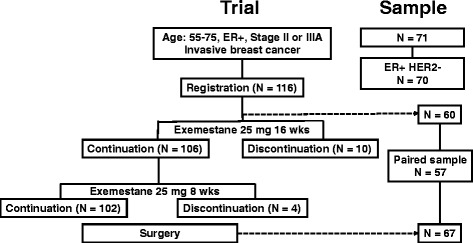
Treatment flow and sample recruitment for JFMC34-0601, a multicenter prospective neoadjuvant exemestane study

The design of the clinical trial JFMC34-0601, a multicenter prospective neoadjuvant exemestane study, was previously reported (Registration number: UMIN C000000345; Fig. 1) [9, 10]. Briefly, the eligibility criteria included the followings: age, 55–75 years; positive ER status; and stage II or IIIa invasive breast cancer. ER or progesterone receptor (PgR) were determined positive using immunohistochemistry (IHC) (≥10 % nuclear staining in local laboratories). In addition, the study treatment included 25 mg/day exemestane for 16 weeks with an 8-week extension, according to the clinical assessment of therapeutic response. Patients with PD during the treatment were withdrawn from the study. At week 24, all the patients underwent surgery. Clinical response was assessed based on the Response Evaluation Criteria in Solid Tumors criteria version 1.0 by caliper measurement and ultrasound, as previously reported [9]. Pathological response was assessed using the following modified criteria, as reported by Miller et al. [11] complete response when there was no evidence of malignant cells at the original tumor site, partial response when histological decrement in cellularity and/or increment in fibrosis was detected, and no response when no changes detected. Pretreatment biopsy and post-treatment surgical tissue specimens were retrieved for this study. The study protocol was approved by the institutional ethics committee at School of Medicine, Kyoto University (Number G-240). Written informed consent was obtained from all patients.

### Immunohistochemistry

**Fig. 2:**
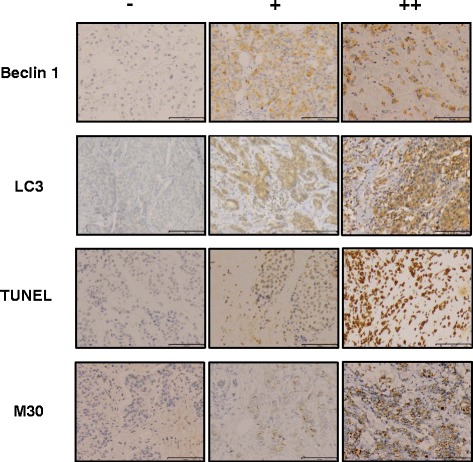
Immunohistochemistry for the autophagy-associated markers beclin 1 and LC3 and apoptosis-associated markers TUNEL and M30. The cytoplasmic staining of beclin 1, LC3, and M30 and nuclear staining of TUNEL were assessed (Scale bar = 100 μm). Weak and strong staining was regarded as positive. -: negative staining, +: weak staining, ++: strong staining

IHC evaluation of ER, PgR, and Ki-67 was centrally performed and assessed by three independent pathologists, as previously reported [9]. Immunostaining was performed using a Histofine Kit (Nichirei, Tokyo, Japan). Ki-67 was evaluated using a 1:100 antibody dilution (Clone MIB-1; Dako, Glostrup, Denmark). The Ki-67 labeling index (LI) was determined by counting 500–1000 tumor cells at the hot-spot sites. The overall average score of Ki67 was also determined in order to further explore the association with stromal beclin 1 immunoreactivity by counting 500–1000 cells in the representative fields of the specimens [12]. ER and PgR immunoreactivity was scored by central assessment according to Allred’s procedure [13], which is used in the analyses of this study. HER2 status was determined using the HercepTest (Dako). In addition, a positive HER2 status was defined as either 3+ or 2+ with confirmed c-erbB2 gene amplification using the FISH test. Apoptosis-associated markers were evaluated using TUNEL (*In Situ* Cell Death Detection Kit; Roche Diagnostics, Mannheim, Germany) and M30 CytoDEATH (1:100; Roche Diagnostics). Autophagy-associated markers were immunostained using the anti-beclin 1 antibody (1:250; NB500-249; Novus Biologicals, CO, USA) and anti-LC3 antibody (1:200; PM036; MBL, Nagoya, Japan), which reacts with LC3A, LC3B and LC3C and detects both LC3-I and LC3-II. The cytoplasmic immunoreactivity of beclin 1, LC3, and M30 and nuclear reactivity of TUNEL were all assessed using pre- and post-treatment tissue specimens (Fig. 2). The positive staining was defined as + (weak staining) or ++ (strong staining). The positive rate of each marker was assessed as positive cells per total cells.

### Statistical analysis

Statistical analyses were performed using the Wilcoxon’s paired test for comparisons between pre- and post-treatment levels of autophagy- and apoptosis-associated markers. The Mann–Whitney test was used for comparing Ki-67 LI between patients with and without stromal beclin 1 expression. The *χ*^2^-test and logistic regression analysis were used to determine the association between markers and clinical and pathological responses. All analyses were performed using the JMP Ver.8.0.1 software package (SAS Institute, Inc., Cary, NC, USA). All p values were two-sided, and *p* < 0.05 was considered statistically significant. All graphs were generated using the GraphPad Prism ver. 5.0.4 software (GraphPad Software, San Diego, CA, USA).

## Results

### Exemestane increased beclin 1 and LC3 but not TUNEL and M30 in carcinoma cells

Tissue specimens were obtained from 71 of the 116 patients in the JFMC34-0601 study. One patient with ER-positive and HER2-positive breast cancers was excluded from the study, leaving 70 patients for 60 core biopsies and 67 resection samples (Table 1). Fifty-seven cases were paired (Fig. 1). The cytoplasmic staining of beclin 1, LC3, and M30 and nuclear staining of TUNEL were assessed (Fig. 2). Both weak (+) and strong (++) immunoreactivity was regarded as positive since, if the positive staining is limited to the strong immunoreactivity, the positive rate became too low. Due to the limited availability of tissue slides, there were some cases in which some markers could not be evaluated (Additional file 1: Table S1).

**Table 1:** Clinical background of the patients

Factor		*N*
Total		70
Age	50–59	12
	60–69	37
	70–79	21
T	2	70
	3	0
N	0	52
	1	18
Clinical Stage	IIA	52
	IIB	18
	IIIA	0
ER	+	70
	-	0
PgR	+	64
	-	6
HER2	+	0
	-	70

**Fig. 3:**
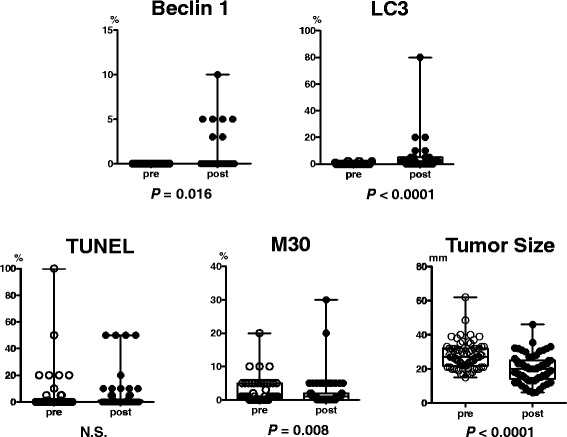
Changes in expression of each marker in cancer cells by treatment. In cancer cells, the autophagy-associated markers, beclin 1 and LC3, increased (*p* = 0.016 and < 0.0001, respectively), whereas M30 decreased (*p* = 0.008) and TUNEL remained unchanged. A decrease in tumor size was also shown (*p* < 0.0001). The y-axis for the markers indicates a positive rate of each marker, which was assessed as positive cells per total cells. Data are shown in box-whisker with dot plots. Horizontal bars in the box-and-whisker plots indicate min–max and 1^st^, 2^nd^, and 3^rd^ quartiles

Beclin 1 and LC3 increased in carcinoma cells following exemestane treatment in 7 (14 %) of 49 patients and 17 (52 %) of 33 patients, respectively. These increases were statistically significant (beclin 1, *p* = 0.016; LC3, *p* < 0.0001). However, M30 decreased after the treatment (*p* = 0.008, *N* = 47) and the value of TUNEL remained unchanged (*N* = 53, Fig. 3). Baseline status of the markers and changes after the treatment were not significantly correlated with clinical and pathological responses of the patients to the treatment (data not shown).

### Baseline beclin 1 in stromal cells was significantly associated with clinical and pathological responses to neoadjuvant endocrine therapy

Stromal cells have been considered to play pivotal roles in cancer treatment and therefore, we evaluated the association between the status of autophagy-associated markers in the stromal cells and treatment responses of the patients. No tumors (0/12) with stromal beclin 1 immunoreactivity in pre-treatment tissues were associated with a pathological response, whereas 41 % (15/37) of tumors without beclin 1 immunoreactivity showed a pathological response (*p* = 0.0081; Table 2). No cases harboring any beclin 1 immunoreactivity in the stromal cells in pre-treatment tissues showed a pathological response and immunoreactivity with > 0 % was therefore defined as positive stromal beclin 1. Consistently, only 25 % (3/12) of the patients with stromal beclin 1 demonstrated a clinical response, whereas 67 % (26/39) of those without beclin 1 expression demonstrated a clinical response (*p* = 0.011; Table 2), suggesting that the status of stromal beclin 1 was associated with the clinical responses of the patients to endocrine treatment. Normal mammary gland epithelia were used as positive control of immunostaining in this study. There were no significant associations detected between other stromal markers at baseline and clinical and pathological responses of the patients.

**Table 2:** Stromal expression of beclin 1 and clinical and pathological response

		Clinical response		Pathological response	
		Non-responder	Responder	Non-responder	Responder
Stromal beclin 1	+	9	3	12	0
	-	13	26	22	15
	*p* value	0.011		0.0081	

In order to further explore the possible factors associated with responses to endocrine treatment, we also performed logistic regression analyses of ER, PgR, Ki-67, and stromal beclin 1 status. The status of stromal beclin 1 immunoreactivity did predict the clinical and pathological responses (*p* = 0.01 for clinical response and 0.0013 for pathological response; Table 3) of the patients while all other factors not. Because all factors other than stromal beclin 1 had p values > 0.2, we did not perform a multivariate analysis for prediction of the treatment response.

**Table 3:** Logistic regression analysis for clinical and pathological response

	Clinical response			Pathological response		
Factors	OR	95 % CI	*p*	OR	95 % CI	*p*
ER	0.76	0.077–7.6	0.81	0.56	0.03–6.2	0.65
PR	1.43	0.24–9.8	0.7	0.52	0.05–3.6	0.52
Ki-67 LI (hot spot)	1.0	0.15–6.5	0.98	2.1	0.26–25	0.49
Stromal beclin 1 (− vs +)	6.0		0.01	140000		0.0013

### Baseline beclin 1 in stromal cells was significantly associated with increased carcinoma cell proliferation

**Fig. 4:**
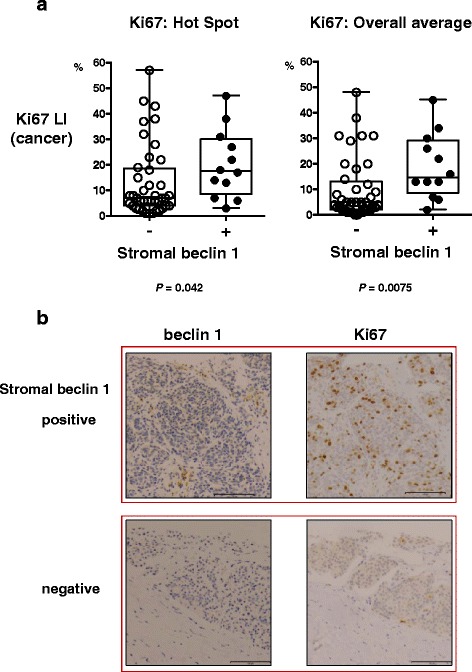
**a** Stromal beclin 1 expression and Ki-67 LI in cancer cells. Tumors with stromal beclin 1 expression revealed higher Ki-67 LI than those without the expression (*p* = 0.042 for hot spot Ki67 and *p* = 0.0075 for overall average Ki67). Data are shown in box-whisker with dot plots. Horizontal bars in the box-and-whisker plots indicate min–max and 1^st^, 2^nd^, and 3^rd^ quartiles. **b** Representative tissues with or without stromal beclin 1 expression. A tumor with stromal beclin 1 expression (*upper panel*) had higher Ki67 LI than a tumor without the expression (*lower panel*). Beclin 1 and Ki67 were stained in serial sections of two representative tissues. Scale bar = 100 μm

We evaluated the association between stromal beclin 1 positivity and Ki-67 LI in carcinoma cells at baseline. Carcinoma cells in tissues with stromal beclin 1 positivity had higher Ki-67 LI than those without its positivity (*p* = 0.042 for hot spot Ki67 and 0.0075 for overall average Ki67, *N* = 53; Fig. 4a), suggesting the correlation between stromal beclin 1 expression and carcinoma cell proliferation. The representative findings of immunohistochemistry were illustrated in Fig. 4b. A tumor with stromal beclin 1 immunopositivity (upper panel) demonstrated higher Ki67 LI than a tumor without stromal beclin 1 (lower panel).

## Discussion

This study demonstrated that treatment of ER-positive breast cancer with exemestane decreased expression of the apoptosis-associated marker, M30, in carcinoma cells, which is consistent with results of the study reported by Dowsett et al. [3]. However, results of our present study also demonstrated that exemestane increased the expression of autophagy-associated markers in carcinoma cells, suggesting that autophagy was involved in cellular responses to endocrine treatment. Autophagy is one of the cell death mechanisms and therefore autophagy induction itself could reduce breast cancer volume. However, results of our present study did not necessarily provide any correlation between the increases of autophagy-associated markers and therapeutic responses to treatment of the patients. In cell lines, autophagy inhibition has been reported to sensitize carcinoma cells to steroidal aromatase inhibitors [14, 15]. In addition, ER-positive carcinoma cells with acquired antiestrogen resistance have been reported to overexpress glucose-regulated protein 78 (GRP 78), which stimulated pro-survival autophagy, suggesting that autophagy serves as one of the resistance mechanisms for endocrine therapy [16]. Therefore, at least at this juncture, autophagy alone may not be involved in the volume loss of tumors. In addition, underlying mechanisms for autophagy induction by exemestane still remain unknown but GRP 78 has been reported to be up-regulated by the selective estrogen receptor down-regulator and therefore, GRP 78 could be one possible mechanism for autophagy induction by endocrine therapy [17]. It is important to determine whether carcinoma cells with autophagy-associated markers are sensitive or resistant to endocrine therapy in breast cancer tissues. Therefore, we planned to follow long-term patient outcomes to examine if an induction of autophagy-associated markers in cancer cells is indeed associated with better or worse patient outcomes.

Results of our present study also demonstrated that stromal beclin 1 positivity at baseline was associated with poor clinical therapeutic response to exemestane. Autophagy in stromal cells has been reported to protect carcinoma cells from cell death [18–20]. Autophagy in cancer-associated fibroblasts induced by a loss of caveolin-1, did protected carcinoma cells from cell death through upregulation of TP53-induced glycolysis and apoptosis regulator in cancer cells [18]. Consistently, the absence of stromal caveolin-1 expression has been also reported to be associated with poor clinical outcome of the patients [19, 20]. In addition, autophagic stromal cells were also reported to fuel carcinoma cells with recycled nutrients, such as pyruvate and lactate, resulting in an adverse clinical outcome of breast cancer patients [20]. Results of our present study also demonstrated that tumors with stromal beclin 1 had higher carcinoma cell proliferation, which is also consistent with the hypothesis above. However, further studies such as a prospective study with a larger number of the cases are required to explore stromal expression of beclin 1 as a predictor of endocrine responsiveness.

The status of baseline stromal beclin 1 was significantly correlated with a poor clinical response to exemestane but that of stromal LC3 not, indicating that beclin 1 and LC3 could be differently regulated. Beclin 1 is a coiled-coil myosin-like BCL2-interacting protein involved in membrane trafficking and initiation and nucleation of the phagophore, whereas LC3 is a ubiquitin-like protein involved in elongation and closure of the autophagosome [21]. LC3 is degraded by autophagy and disappearance of total LC3 could also be a good indicator of autophagic flux [22]. Therefore, an involvement in different autophagic phases and different turnovers could lead to the possible discrepancy between beclin 1 and LC3 expressions. p62 is also involved in autophagy-dependent elimination of different cargos including ubiqutinated protein aggregates and constantly degraded by autophagy and, therefore, useful for measuring autophagic flux. However, due to the limited availability of tissue slides, the assessment of p62 expression could not be performed in this study and further investigations are required for clarification of the role of autophagy in endocrine treatment.

In this study, the status of ER and PgR was not necessarily associated with clinical and pathological responses to exemestane, although ER is a key biomarker for endocrine treatment. This could be due to narrow ranges of ER and PgR status of the tumors evaluated in this study. Indeed, more than 95 and 70 % of tumors in this NAE study had relatively high ER and PgR expressions, respectively, with Allred’s score ≥ 6. Results of our present study did not show a predictive value of Ki67 LI for responses to neoadjuvant endocrine therapy in concordance with previous studies [11, 23].

There are some limitations in this study. One of the limitations is the relatively small sample size. Specimen availability for this study was limited, and surgical specimens from patients who had discontinued treatment were not obtained. Another limitation is that only exemestane was used as an aromatase inhibitor. It remains unknown if nonsteroidal aromatase inhibitors could show similar results in breast cancer tissues.

## Conclusions

Beclin 1 and LC3 immunoreactivity in carcinoma cells increased following exemestane treatment, and the stromal beclin 1 at baseline was associated with poor clinical and pathological responses to exemestane in this study. A larger study is definitively required to confirm our results but an elucidation of stromal involvement in response to endocrine treatment could possibly lead to a novel treatment strategy which targets stromal cells for further improvement of clinical outcomes of breast cancer patients.

### Ethics approval and consent to participate

The study protocol was approved by the institutional ethics committee at School of Medicine, Kyoto University (Number G-240). Written informed consent was obtained from all patients.

### Consent for publication

Not applicable.

### Availability of data and materials

The dataset supporting the conclusions of this article is included within the article (Additional file 1: Table S1).

## Additional file

Additional file 1: Table S1. All the data supporting the conclusion in this paper. (XLS 48 kb)

## Abbreviations

GRP 78: glucose-regulated protein 78
HR: hormone receptor
IHC: immunohistochemistry
LI: labeling index
NAE: neoadjuvant endocrine therapy
PD: progressive disease
PgR: progesterone receptor
